# Isoproterenol-Dependent Activation of TRPM7 Protects Against Neurotoxin-Induced Loss of Neuroblastoma Cells

**DOI:** 10.3389/fphys.2020.00305

**Published:** 2020-04-24

**Authors:** Yuyang Sun, Amrita Kamat, Brij B. Singh

**Affiliations:** ^1^Department of Periodontics, School of Dentistry, University of Texas Health Science Center at San Antonio, San Antonio, TX, United States; ^2^Geriatric Research, Education and Clinical Center, South Texas Veterans Health Care System, University of Texas Health Science Center at San Antonio, San Antonio, TX, United States

**Keywords:** Mg^2+^ influx, TRPM7 activation, β-AR, cell survival, apoptosis, neurodegeneration

## Abstract

Neuronal function and their survival depend on the activation of ion channels. Loss of ion channel function is known to induce neurodegenerative diseases such as Parkinson’s that exhibit loss of dopaminergic neurons; however, mechanisms that could limit neuronal loss are not yet fully identified. Our data suggest that neurotoxin-mediated loss of neuroblastoma SH-SY5Y cells is inhibited by the addition of β-adrenergic receptor (β-AR) agonist isoproterenol. The addition of isoproterenol to SHSY-5Y cells showed increased Mg^2+^ influx and cell survival in the presence of neurotoxin especially at higher concentration of isoproterenol. Importantly, isoproterenol potentiated transient receptor potential melastatin-7 (TRPM7) channel activation that leads to an increase in intracellular Mg^2+^ levels. The addition of 2APB, which is a known TRPM7 channel blocker, significantly decreased the TRPM7 function and inhibited isoproterenol-mediated protection against neurotoxins. Moreover, neurotoxins inhibited TRPM7 expression and function, but the restoration of TRPM7 expression increased neuroblastoma cell survival. In contrast, TRPM7 silencing increased cell loss, decreased Mg^2+^ homeostasis, and inhibited mitochondrial function. Moreover, isoproterenol treatment prevented neurotoxin-mediated loss of TRPM7 expression and inhibited Bax expression that induces cell survival. These effects were dependent on the neurotoxin-induced increase in oxidative stress, which inhibits TRPM7 expression and function. Together, our results suggest a positive role for β-AR in activating TRPM7 channels that regulate Mg^2+^ homeostasis and are essential for the survival of SH-SY5Y cells from neurotoxin.

## Introduction

Parkinson’s disease (PD) is an age-related movement disorder that is mainly due to selective degeneration of nigrostriatal dopaminergic (DA) neurons (Venderova and Park, 2012). The major clinical symptoms observed in PD are rigidity, bradykinesia, and resting tremor, which are caused by the deficiency in neurotransmitter dopamine-mediated signaling (Javitch et al., 1985; Venderova and Park, 2012). Additionally, non-motor symptoms are also present in these patients, including cognitive and autonomic functions, olfactory, sleep, mood disorders, and gut physiology, which may not be directly due to the loss of other neurons (Chen et al., 2019). Although several mutations have been identified, majority of them (90%) of these PD cases identified, are idiopathic or sporadic in nature, and only a small percentage of patients exhibit genetic mutations, suggesting that exogenous factors makes these DA neurons vulnerable (Surmeier et al., 2017). Environmental factors such as neurotoxins have been one of the major inducers of PD, thus toxin-induced animal models have been crucial in elucidating the pathophysiology of PD. 1-Methyl-4-phenyl-1,2,3,6-tetrahydropyridine (MPTP) is an exogenous neurotoxin that induces Parkinson’s like symptoms in humans, monkeys, and other small animals. MPTP is metabolized by monoamine oxidase type B (MAO-B) that is present in microglia/astrocytes cells, into 1-methyl-4-phenyl-pyridinium ion (MPP^+^), which selectively destroys the nigrostriatal DA neurons (Javitch et al., 1985). Although protection against neurotoxins-mediated loss of DA neurons has been a major focus in preventing PD, the exact mechanisms involved in DA neuronal loss are not known. Recent studies have shown that imbalance in divalent cations that could lead to ER/oxidative stress and/or mitochondrial dysfunction could contribute to PD (Tatton and Olanow, 1999; Dawson and Dawson, 2003; Selvaraj et al., 2012; Sukumaran et al., 2018). Moreover, divalent cations such as calcium (Ca^2+^) or magnesium (Mg^2+^), which modulates cellular processes such as cell proliferation, mitochondrial function and energy metabolism, gene regulation, and synthesis of biomacromolecules (Selvaraj et al., 2012) has gained much attention as this could be a possible target for understanding PD.

Lower Mg^2+^ concentrations have been observed in the brain samples of PD patients as compared with non-PD subjects (Uitti et al., 1989; Bocca et al., 2006). Importantly, these decreases in Mg^2+^ concentrations were present in the substantia nigra region/mid brain region especially nucleus accumbens and the caudate nucleus (Uitti et al., 1989; Bocca et al., 2006). Furthermore, intracellular Mg^2+^ concentrations showed a significant correlation with the severity of PD and the extend of the disease phenotype observed (Uitti et al., 1989). Consistent with these reports, mice that had decreased Mg^2+^ concentrations exhibited an increase in the loss of neurotoxin-mediated cell death, especially in the DA neurons (Muroyama et al., 2009). Similarly, animals treated with another neurotoxin, 6-hydroxydopamine also exhibited decreased intracellular Mg^2+^ concentrations when compared with control mock-treated mice (Sturgeon et al., 2016). Although the channels that modulate Mg^2+^ influx are not well identified, Transient receptor potential Melastatin 6 and 7 (TRPM 6 and 7) channels are the main channels that modulate intracellular Mg^2+^ levels in various cells. Interestingly, *TRPM7* has been observed to be mutated in Guamanian ALS/PD patients (Hermosura et al., 2005) and TRPM7 expression is observed to be blunted in PD patients along with a similar decrease in neurotoxin models of PD (Sun et al., 2015). Similarly, TRPM7 mutants in zebrafish have decreased DA neurons (Decker et al., 2014), suggesting that changes in the Mg^2+^ influx could induce neurodegeneration. Consistent with this observation, decreased Mg^2+^ intake induced DA neuron loss, whereas Mg^2+^ supplementation prevented neurotoxin-mediated decrease in DA neurons (Oyanagi and Hashimoto, 2011; Sun et al., 2019). These results suggest that TRPM7-mediated regulation of intracellular Mg^2+^ could promote neuronal survival, however, its regulation, specifically TRPM7 activation in DA cells is not fully identified.

Increased intracellular levels of cAMP have also been shown to increase DA neurons survival and protect them from MPP^+^-mediated degeneration (Scarpace et al., 1991; Hartikka et al., 1992). Importantly, β-adrenergic receptors (β_1_-, β_2_-, and β_3_-AR subtypes) mediate the action of catecholamines via the classical adenylyl cyclase/cAMP/protein kinase A (PKA) cascade to modulate important biological responses (Hishida et al., 1992). Previous studies utilizing small groups of PD patients have demonstrated that co-administration of salbutamol (a β_2_-AR agonist) with levodopa helps reduce parkinsonian symptoms (Alexander et al., 1994; Uc et al., 2003). Furthermore, longitudinal analyses of PD incidents in Norway demonstrated that the use of salbutamol is associated with a decreased risk of developing PD while treatment with β-AR antagonist (beta-blocker) propranolol increased the risk of suffering from PD (Mittal et al., 2017). Similarly, β_2_-AR agonist clenbuterol reduced the levels of α-synuclein protein and protected against neurotoxin-induced degeneration of dopaminergic neurons (Mittal et al., 2017). Importantly, TRPM7 has been shown to be activated by β-AR in non-excitable cells, however, is similar mechanisms are observed in DA neurons is not yet defined. Thus, the purpose of this study was to establish if TRPM7 activation via β_2_-AR agonist modulates neuronal survival. Our data suggest that β-AR agonist protects against neurotoxin-mediated loss of neuroblastoma cells, which was mediated through TRPM7. β-AR agonist potentiated TRPM7 function and maintained Mg^2+^ homeostasis that is essential for the survival of neurotoxin-induced loss of neuroblastoma SH-SY5Y cells. Furthermore, knockdown of TRPM7 abolished the protective effect of β-AR agonist, whereas TRPM7 overexpression increased intracellular Mg^2+^ levels and prevented MPP^+^-induced cellular death. These results suggest that β-AR-mediated activation of TRPM7 could be essential in the survival of neurons especially in neurotoxin-induced degeneration.

## Materials and Methods

### Cell Culture and Chemicals

Neuroblastoma cells (SH-SY5Y) were previously obtained from the American Type Culture Collection (Manassas, VA, United States), which were cultured as suggested and differentiated into dopaminergic like cells using retinoic acid (10 μM) for 7 days as previously described (Bollimuntha et al., 2005) prior to be used for all the experiments. The chemicals used were: 1-Methyl-4-phenylpyridinium, 2-Aminoethoxydiphenyl borate, Isoproterenol (+)-bitartrate salt which were purchased from Sigma-Aldrich. ISO was freshly prepared and dissolved in PBS and used for the experiments.

### Transient Transfections and Cell Viability Assays

For the silencing of TRPM7 expression, shRNA plasmids that specifically targets the coding sequence of human TRPM7 was obtained from Origene (Rockville, MD, United States). All transfections were transient and differentiated SH-SY5Y cells were used for all experiments using lipofectamine as previously described (Sun et al., 2018). For TRPM7 overexpression, full length HA-TRPM7 plasmids was used to transiently overexpress TRPM7 in these cells. Briefly, 5 μg of the plasmid DNA was used to transform differentiated SH-SY5Y cells using Lipofectamine in the Opti-MEM medium for 24 h as indicated. To measure cell viability SH-SY5Y cells were trypsinized, counted, and seeded equally on 96-well plates at a density of 0.5 × 10^5^ cells/well. The cultures were grown for 24 h with appropriate treatments as labeled in the figure and cell viability under various conditions was measured using the MTT regents as previously described by us (Selvaraj et al., 2012). Cell viability was expressed as a percentage of the control (untreated) when compared with neurotoxin treatment. The methods described here are modified from our previous publication (Sun et al., 2018).

### Electrophysiology

For patch-clamp experiments, differentiated SH-SY5Y cells were grown on glass coverslips and single coverslips were placed in the recording chamber. The cells perfused with an external Ringer’s solution that has the following composition (in mM): NaCl, 145; CsCl, 5; MgCl_2_, 1; CaCl_2_, 1; Hepes, 10; Glucose, 10; pH 7.3 (NaOH). Whole-cell currents were recorded using an Axopatch 200B (Axon Instruments, Inc.) (Singh et al., 2000). The patch pipette used for each experiment had a resistance between 3 and 5 MΩ, which was measured after filling the standard intracellular solution, which contained the following (in mM): Cesium methanesulfonate, 150; NaCl, 8; Hepes, 10; EDTA, 10; pH 7.2 (CsOH). After whole cell configuration was established, the voltage ramp protocol was initiated that ranged from −100 mV to +100 mV and 100 ms duration were delivered at every 2 s intervals formed. Currents observed in each condition were recorded at 2 kHz, digitized followed by analysis using the pClamp 10.1 software that was used for data acquisition as well. The data presented is from an average of 6–10 cells in each condition. Basal leak currents were subtracted from the final currents (when current reach the peak) and average currents are shown. The methods used for this study are taken from our previous publication (Sun et al., 2018).

### Magnesium/Calcium Imaging

For Imaging experiments, differentiated cells that were grown on glass bottom coverslips were incubated with 2 μM Mag-Fura 2-AM (Invitrogen) for the measurement of intracellular Mg^2+^ or with Fura-2 (Molecular Probes for 45 min) for the measurement of intracellular Ca^2+^. After loading cells were washed twice with SES (Standard External Solution that includes: 10 mM HEPES, 120 mM NaCl, 5.4 mM KCl, 1 mM MgCl_2_, 10 mM glucose, pH 7.4) buffer. For fluorescence measurements, the fluorescence intensity of Fura-2 or Mag-FURA-loaded cells was monitored with a CCD camera-based imaging system linked with an Olympus XL70 inverted fluorescence microscope. Fluorescence traces from individual cells imaged were obtained and the data shown represent [Mg^2+^]_*i*_ or Ca^2+^ values that are average from at least 30–40 cells. Also, the data presented are representative of at least 3–4 individual experiments performed in duplicate. Mg^2+^ or Ca^2+^ concentrations in individual cells were estimated by evaluating the 340/380 ratio as described before in (Sun et al., 2018).

### Western Blot Analysis

Cell lysates (differentiated SH-SY5Y cells) under different conditions (as labeled in the figures) were obtained using NP40 or 0.5% SDS treatment for 15 min on ice followed by centrifugation at 10,000 × *g* for 15 min at 4°C. Protein concentrations from all treatments as labeled in the figures were evaluated using the Bradford reagent (Bio-Rad) and 25 μg of total lysates from individual samples were resolved on NuPAGE 4–12% Bis-Tris gels. Western blotting were performed using specific antibodies (Singh et al., 2000; Selvaraj et al., 2009). The antibodies used were the following monoclonal or polyclonal antibodies: anti-TRPM7 (Abcam, MA; Cat# 109438; 213 kDa; Dilution in 1:500), anti-Bcl_2_ (Cell Signaling, MA; Cat# 209039; Dilution used were 1:1000), anti-Bax (Cell Signaling, MA; Cat# 5023; Dilution used was 1:1000), anti-β-Actin (Cell Signaling, MA; Cat# 4970, at 1:2000 dilution) and anti- β_2_-AR (Abcam, MA; Cat# ab36956; Dilution used was 1:1000). The methods described here are taken from our previous publication (Sun et al., 2018).

### Mitochondrial Membrane Potential

Rhodamine 123 was used to measure the Mitochondrial transmembrane potential as described in Selvaraj et al. (2009). To quantify the membrane potential, fluorescence signals observed in different conditions were measured (excitation wavelength used was 488 nm and an emission wavelength used was 510 nm) using a fluorescence microplate reader (biotex) and plotted as percentage.

### Statistical Analysis

Origin 9.0 (Origin Lab) was used for all data analysis. Statistical significance was established either using Student’s *t*-test or one-way ANOVA (*post hoc* using Tukeys or Fisher test when compared between more than 2 variables). All values indicated in the figure are shown as means ± SEM or ± SD as stated in the figure legends. *p*-value are also indicated and 0 < 0.05 or lower were considered significant.

## Results

### β-Adrenergic Receptor Agonist Protects Against Neurotoxin-Dependent Loss of Cells

**FIGURE 1:**
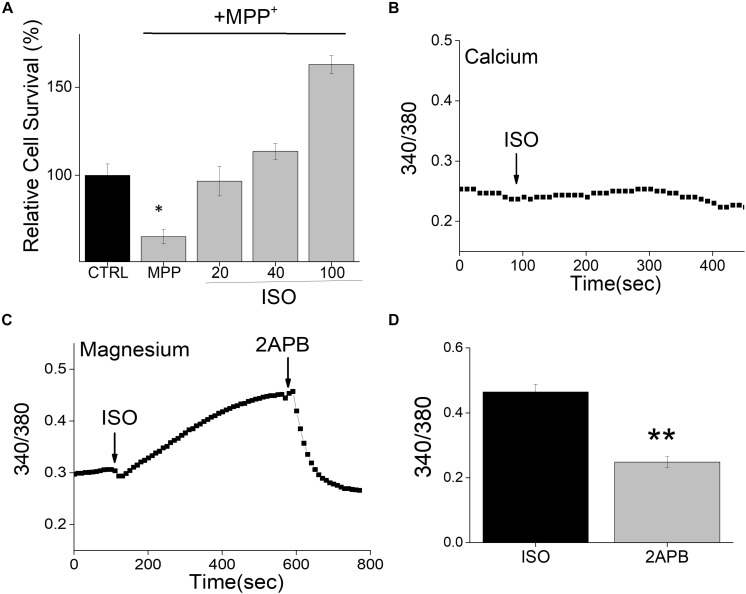
Isoproterenol treatment induces survival of neuroblastoma cells: **(A)** Cell survival under conditions as labeled was performed using MTT assays on control and MPP^+^ (500μM for 24 h) treated SH-SY5Y cells. The conditions used were control (mock treatment), ISO 20, 40, and 100 μM, which was added 15 min prior to the addition of MPP^+^. Individual columns show the means ± SD of 5 separate experiments performed in triplicates (*p < 0.05, **p < 0.01; One-way ANOVA, Tukey *post hoc* test). **(B)** Ca^2+^ imaging in Fura 2 loaded cells was performed by the application of ISO (100 μM) in normal SES (1 mM Ca^2+^, 1 mM Mg^2+^) solution in SH-SY5Y cells. **(C)** Mg^2+^ imaging was performed using mag-Fura in normal SES (1 mM Ca^2+^, 1 mM Mg^2+^) solution using differentiated SH-SY5Y cells. Application of ISO (100 μM) in the external solution induces Mg^2+^ influx and addition of 2APB (100 μM) inhibits Mg^2+^ influx in these cells. **(D)** Quantification (mean ± SE) of intracellular Mg^2+^ concentrations are also included as bar graph.

Neurotoxins, such as 1-methyl-4-phenyl-1,2,3,6-tetrahydropyridine (MPTP) metabolite MPP^+^, induce a loss of DA neurons in most vertebrate animals including sub-human primates lower animals that show Parkinson’s disease (PD) like symptoms (Burns et al., 1983). We thus used differentiated neuroblastoma SH-SY5Y cells that were treated with MPP^+^ to examine the effect of this neurotoxin on its survival. Consistent with our previous results (Sun et al., 2017), addition of neurotoxin, MPP^+^ showed an increase in cell death in differentiated SH-SY5Y cells (Figure 1A). Importantly, pretreatment of SH-SY5Y cells with β-adrenergic receptor (β-AR) agonist, isoproterenol showed a dose-dependent increase in cell survival (Figure 1A). Interestingly, even low doses (20 μM) of isoproterenol (ISO) showed a significant decrease in cell death when compared with cells that were treated with MPP^+^ alone. Furthermore, increase in the doses used for ISO treatment (more than 40 μM) further increased cell survival, which was much higher than control untreated cells in 100 μM of ISO treatment. β-AR activation has been shown to mobilize intracellular Ca^2+^ via the non-canonical cAMP-independent pathway (Galaz-Montoya et al., 2017), thus we evaluated if isoproterenol treatment induces Ca^2+^ entry. However, as shown in Figure 1B, the addition of isoproterenol did not increase cytosolic Ca^2+^ levels in these cells. In contrast, an increase in Mg^2+^ concentration was observed upon addition of isoproterenol, which was significantly decreased upon the addition of 2APB (Figures 1C,D). Together these results suggest that isoproterenol stimulation modulates intracellular Mg^2+^ concentration that could protect against neurotoxin-induced cell death.

**FIGURE 2:**
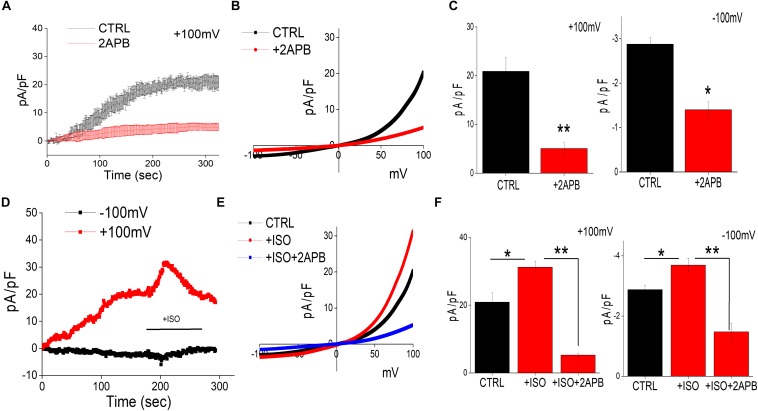
Isoproterenol-Induces TRPM7 activation in neuroblastoma cells: **(A)** Representative trace showing whole cell recording (outward currents) in control cells and cells pretreated with 2APB (100 μM). The external solution used was normal SES solution that had 1 mM Ca^2+^ and Mg^2+^ respectively was used to obtain the currents (outward/inward) at +100 mV/–100 mV in differentiated SH-SY5Y cells. **(B)** IV curves under these conditions (control and bath pre application of 100 μM 2APB) as labeled in the figure were obtained using peak currents. Quantitation of current density at ±100 mV is shown in **(C)**. * Indicate significance (*p* < 0.05) and ** Indicate significance (*p* < 0.01). **(D)** Traces showing whole cell recording in control cells at ±100 mV were recorded followed by stimulation with ISO (bath application of 20 μM ISO, with and without pretreatment of 100 μM 2APB) as labeled in the figure. IV curves shown are acquired when currents reach their peak in each condition and are shown in **(E)** Quantitation of current density at ±100 mV is shown in **(F)**. The columns show the means ± SD of 6 experiments.

Recent studies have shown that intracellular Mg^2+^ concentration is mediated through TRPM6/7 channels (Sun et al., 2019). Whole-cell current recordings were used to further establish the channel identity that is responsible for Mg^2+^ influx. In differentiated SH-SY5Y cells decrease in intracellular Mg^2+^ generated a current, which was both inward and outward rectifying and reversed around zero mV (Figures 2A–C). The properties of the currents were similar as observed with TRPM6/7 channels. 2APB has been previously used to differentiate TRPM6/7 currents as addition of 2APB potentiates TRPM6 function, but inhibits TRPM7 currents (Mishra et al., 2009; Sun et al., 2019). Thus, to differentiate between these two Mg^2+^ channels we further studied the effects of 2-APB that further decreased these currents, suggesting that these currents are mainly through TRPM7 channels (Figures 2A–C). Also, the characteristics of the current observed was consistent with TRPM7 channels, which have been previously reported (Sun et al., 2013). Importantly, addition of isoproterenol further increased TRPM7 currents at both positive and negative membrane potentials, which was again inhibited by the addition of 2APB (Figures 2D–F).

### TRPM7 Expression and Function Modulate Cell Survival in SH-SY5Y Cells

**FIGURE 3:**
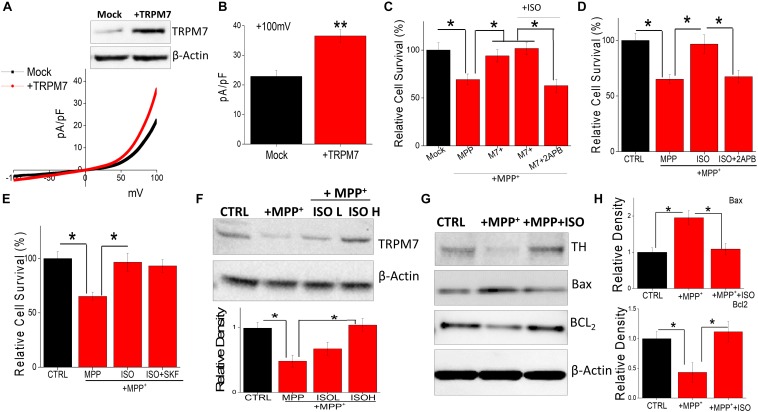
Restoration of TRPM7 expression inhibits neurotoxin-induced loss of SH-SY5Y cells: IV curves in control and TRPM7 overexpressing cells were measured and shown in **(A)**. Western blots were also performed in these cells to look at TRPM7 expression under these conditions. Quantitation of current density at +100 mV is demonstrated in **(B)**. **(C–E)** MTT assays were performed to observe cell survival on TRPM7 overexpressed cells **(C)** and control cells **(D,E)** with and without MPP^+^ (500 μM for 24 h) under various conditions as labeled (+ISO 20 μM, or +SKF 10 μM). The columns show the means ± SD of 5 individual experiments performed in triplicates. (*p < 0.05; One-way ANOVA, Tukey *post hoc* test). Sample from differentiated SH-SY5Y [treated for 24 h with MPP^+^ 500 μM, with and without ISO 20 μM (L) and 40 μM (H)] were resolved and protein expression was evaluated by western blotting, antibodies use is labeled in the figures **(F)**. Quantification of TRPM7 is shown as bar graph. **(G)** Also shows western blots under different conditions, antibodies used are labeled in the figure. **(H)** Shows quantification where the columns represent mean ± SD of 3 independent experiments that were normalized by β-actin expression. (*p < 0.05; **p < 0.01; One way ANOVA, Tukey *post hoc* test).

The data presented thus far suggest that isoproterenol activates TRPM7 currents. Hence, we further investigated this relationship, and cells overexpressing TRPM7 showed increased TRPM7 protein levels, without altering the expression of β-actin, which was used as a loading control (Figure 3A). Furthermore, an increase in the TRPM7 currents was observed in SH-SY5Y cells overexpressing TRPM7 (Figures 3A,B). Importantly, overexpression of TRPM7 inhibited MPP^+^-mediated cell death of neuroblastoma cells (Figure 3C). Moreover, addition of low doses of isoproterenol (20 μM) in TRPM7 overexpressing cells did not increase cell protection any further (Figure 3C). In contrast, inhibition of TRPM7 currents using 2APB further increased MPP^+^-mediated cell death even at low doses (20 μM) of isoproterenol, which was again blocked in TRPM7 overexpressed cells (Figure 3C). Similar results were obtained where MPP^+^-inhibited cell survival of control untreated cells that do not overexpress TRPM7 (Figure 3D). In contrast, ISO treatment (20 μM) blunted the effects of MPP^+^; however, pretreatment with 2APB inhibited ISO-mediated protection in control cells (Figure 3D). Importantly, the addition of non-specific Ca^2+^ channel blocker (SKF 96365) failed to block isoproterenol-mediated protection of differentiated SH-SY5Y cells (Figure 3E). Furthermore, addition of MPP^+^ decreased TRPM7 expression, whereas addition of isoproterenol (even at lower doses, 20 μM) was able to prevent MPP^+^-induced loss of TRPM7 (Figure 3F). We next evaluated the expression of various proteins upon exposure of SH-SY5Y cells to MPP^+^ in the absence or presence of isoproterenol. Importantly, a significant decrease in DA neuron marker [tyrosine hydroxylase (TH)] was observed upon addition of MPP^+^, which was partially rescued upon prolonged treatment with isoproterenol (Figure 3G). Consistent with TH expression, MPP^+^-treatments also significantly increased expression of Bax, which is a pro-apoptotic protein along with a subsequent decrease in Bcl_2_, an anti-apoptotic protein, without any change in actin levels (Figures 3G,H). Furthermore, treatments with isoproterenol decreased the expression of Bax and increased expression of Bcl_2_ (Figures 3G,H), suggesting that TRPM7 expression and function are modulated via isoproterenol, which is essential for the neurotoxin-induced survival of neuroblastoma cells.

**FIGURE 4:**
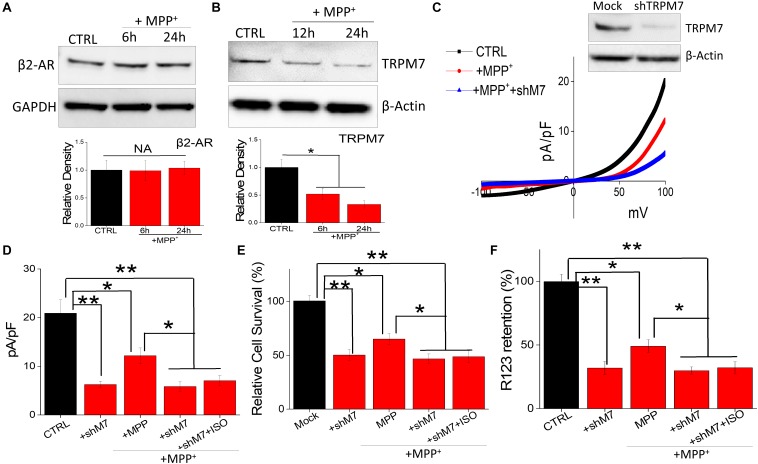
Neurotoxin treatment inhibits TRPM7 expression/function: **(A)** Western blots showing the expression of β_2_-adrenergic receptor **(A)**, TRPM7 **(B)**. β-actin was used as a loading control in CTRL and MPP^+^-treated (500 μM for 24 h) cells. **(C)** IV curve and quantitation of currents observed are shown from control, MPP^+^ treatment (500 μM, 24 h), and shTRPM7 cells. **(D)** The columns represent means ± SD of 6 independent experiments. (**p < 0.01, *p < 0.05; was established using one-way ANOVA, Tukey *post hoc* test). MTT assays **(E)** and mitochondrial transmembrane potential **(F)** were evaluated under various conditions as labeled in the figure. The concentration of ISO used was 20 μM and for MPP^+^ 500 μM was used for each experiment. The columns show the means ± SEM of 4 independent experiments. (**p < 0.01, *p < 0.05).

It is also possible that neurotoxin treatment could alter the expression of β-AR subtype, which could contribute toward cell death. Interestingly, neurotoxin treatment did not alter the expression of β_2_-AR subtype (Figure 4A); whereas loss of TRPM7 protein was observed in MPP^+^-treated neuroblastoma cells (Figure 4B). These results strongly suggest that neurotoxin-mediated loss of neuroblastoma SH-SY5Y cells are dependent on TRPM7 expression. To further establish the role of TRPM7, we transiently knocked down TRPM7 expression in these cells. TRPM7 silencing decreased TRPM7 protein levels, its function, and addition of neurotoxin in TRPM7 silenced SH-SY5Y cells showed a further decrease in TRPM7 activity (Figure 4C). Moreover, TRPM7 silencing, abolished the isoproterenol-induced increase in intracellular Mg^2+^ (Figure 4D). Similarly, the protective effect observed with isoproterenol was also abolished as TRPM7 knockdown prevented cell survival and inhibition of apoptosis by isoproterenol was inhibited (Figure 4E). As mitochondrial membrane potential is critical for cell survival, we used rhodamine 123 to elucidate the role of TRPM7 activity in regulating neurotoxin-mediated loss of mitochondrial membrane potential. As expected, MPP^+^ treatment resulted in a reduction of mitochondrial membrane potential as compared with control untreated cells (Figure 4F). Moreover, silencing of TRPM7 further decreased MPP^+^-mediated mitochondrial membrane potential, which was not restored even upon isoproterenol treatment (Figure 4F). These results further show that isoproterenol-mediated protection is dependent on TRPM7 expression and function.

### Neurotoxin Treatment Induces Reactive Oxygen Species That Modulates TRPM7 Expression and Function

**FIGURE 5:**
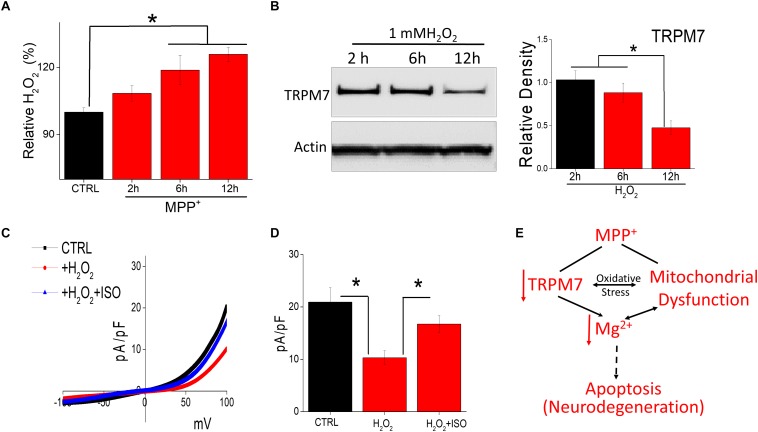
Oxidative stress induced by neurotoxin treatment inhibits TRPM7 expression/function: **(A)** Relative H_2_O_2_ release was evaluated under various conditions (MPP^+^-treated 500 μM) in SH-SY5Y cells. The columns show the means ± SEM of 4 experiments. (*p < 0.05, **p < 0.01; One-way ANOVA, Tukey *post hoc* test). **(B)** Western blots showing the expression of TRPM7 and loading control β-actin in CTRL and H_2_O_2_-treated (1 mM) in SH-SY5Y cells. **(C)** IV curves of TRPM7 currents under conditions as labeled in the figure, which are quantitation as current density at ±100 mV [mean ± SD of 6 experiments (**p < 0.01)] are shown in **(D)**. **(E)** Proposed model for the role of TRPM7 in MPP^+^ mediated cell death.

Oxidative stress has also been suggested to be a cause for the degeneration of dopaminergic neurons (Bollimuntha et al., 2011). Thus, H_2_O_2_ generation in neurotoxin-treated cells was evaluated. Addition of MPP^+^ showed a time dependent increase in intracellular H_2_O_2_ generation (Figure 5A). To evaluate the consequence of endogenous H_2_O_2_, we evaluated the expression and function of TRPM7 channels. MPP^+^ treatment significantly decreased TRPM7 protein level within 12 h of exogenous H_2_O_2_ treatment, without any noticeable change in the actin levels (Figure 5B). Consistent with western blot data, exogenous H_2_O_2_ application in SH-SY5Y cells decreased TRPM7 activity, which was reversed upon isoproterenol treatment (Figures 5C,D). Consistent with TRPM7 activity, the cell death was also significantly higher in H_2_O_2_ treated cells, which was further reversed upon isoproterenol treatment (data not shown). These findings implicate that H_2_O_2_ accumulation is observed upon neurotoxin-treatment that decreases TRPM7 expression thereby decreasing intracellular Mg^2+^ concentration essential for the survival of neuroblastoma cells.

## Discussion

Neurotoxin treatment has been well used as a model for PD and using this *in vitro* model we have here established the significance of β−AR-mediated activation of TRPM7 in the loss of neuroblastoma cells. We have previously shown that Mg^2+^ homeostasis prevents neurotoxin-induced cell death (Sun et al., 2019), however, the mechanisms for TRPM7 activation are not known. Importantly, increasing Mg^2+^ concentration has been shown to protect against neurotoxin-induced loss of dopaminergic cells (Hashimoto et al., 2008; Muroyama et al., 2009), suggesting that Mg^2+^ influx leads to the survival of dopaminergic neurons. Mg^2+^ is a divalent cation that regulates physiological processes such as mitochondrial function, ATP modulation, and cell survival (Romani, 2011; Zhang et al., 2012). The data presented here indicate that loss of Mg^2+^ homeostasis (either by the addition of neurotoxin or by artificially silencing TRPM7 expression) results in a loss of mitochondrial membrane potential, which leads to apoptosis. Importantly, loss of mitochondrial integrity facilitates the translocation of Bax protein to the mitochondria activating the mitochondrial transition pore that induces apoptosis-mediated cell death (Enari et al., 1998). Apoptosis is the main mechanism that promotes the loss of DA neurons (Selvaraj et al., 2009; Venderova and Park, 2012). Consistent with these published studies, our results using differentiated SH-SY5Y cells also showed that neurotoxin treatment leads to alteration in protein expression that are involved in apoptosis. Importantly, Bax, a pro-apoptotic protein, was decreased upon the addition of isoproterenol; whereas, Bcl_2_ (a member of anti-apoptotic proteins) expression was sequestered upon neurotoxin treatment (but reversed upon the addition of isoproterenol) that could initiate mitochondrial-mediated cell death.

One of the important findings presented here was that TRPM7 is the major ion channel that modulates Mg^2+^ homeostasis in neuroblastoma cells. Our results also show that MPP^+^ or H_2_O_2_ treatments, which induces ROS, significantly decrease TRPM7 protein levels. Although the exact mechanism as how TRPM7 expression is decreased is not known, ROS has been shown to alter gene expression. This neurotoxin-mediated loss of TRPM7 expression further decreases TRPM7 activity thereby decreasing intracellular Mg^2+^, which leads to the loss of neuroblastoma cells. Although the mechanisms involved in TRPM7-mediated protection of neuronal cells is not clear, an increase in pro-apoptotic proteins along with a decrease in ATP levels could be the major reason for the loss of neuroblastoma cells. In addition, the presence of ROS could increase the release of Zn^2+^ from TRPM7 vesicles which could also contribute toward neurodegeneration.

Importantly, the decrease in TRPM7 expression was specific, since no change in actin levels were observed upon neurotoxin-treatment. Consistent with these studies a decrease in TRPM7 expression has also been shown in PD patients and in the samples from neurotoxin-induced substantia nigra pars compacta regions (Sun et al., 2019), which further suggests that loss of TRPM7 could lead to neurodegeneration. Similarly, mutations in TRPM7 has been reported in some familial PD patients (Hermosura et al., 2005), and although the expression and/or function of TRPM7 was not identified in this study, a decrease in TRPM7 expression and/or function could be the reason for the observed neuronal loss. Intracellular Mg^2+^ levels have been shown to rescue cell growth and increase viability (Schmitz et al., 2003), and as TRPM7 down-regulation further leads to a decrease in intracellular Mg^2+^, it could be suggested that loss of Mg^2+^ homeostasis could be the reason for the loss of dopaminergic cells; however, activation of TRPM7 is not known. Importantly, our data showed that addition of β-adrenergic receptor agonist, isoproterenol, even at low doses that are physiologically relevant significantly increased TRPM7 activity and inhibited neurotoxin-mediated cell death. Moreover, isoproterenol-mediated activation of TRPM7 restored Mg^2+^ homeostasis in neuroblastoma cells. Another important aspect of this study was that the concentration of isoproterenol used was much lower that will limit any off target effects. Furthermore, ISO-mediated protection was dependent on TRPM7 expression, as TRPM7 silencing cells failed to show increased cell survival even in the presence of isoproterenol. Moreover, apoptosis was increased in cells treated with siTRPM7 even in the presence of isoproterenol. In contrast, restoration of TRPM7 expression increased intracellular Mg^2+^, inhibited apoptosis, and promoted cell survival. These results further emphasize the importance of TRPM7 as expression of other Mg^2+^ transporters were unable to overcome the loss of TRPM7. β-AR agonists, have been shown to increase the survival of DA neurons (Peterson et al., 2014; Sun et al., 2018), however, the mechanism is not clear. Based on our findings, we postulated that β-AR agonist activates TRPM7 channel activity that could modulate the survival of DA cells/neurons. Importantly, TRPM7 levels were decreased in the presence of neurotoxins, but pretreatment with β-AR agonists even at low doses increased TRPM7 levels, which restored Mg^2+^ homeostasis thereby inhibiting cell death. Although the exact mechanism involved in isoproterenol-mediated protection of dopaminergic neurons is not fully established, it could inhibit reactive oxygen species (ROS) formation, which regulates TRPM7 expression and maintains appropriate Mg^2+^ levels in dopaminergic cells. Importantly, a recent study has shown that addition of low concentrations of β-AR agonist inhibited the LPS-induced production of inflammatory mediators, such as ROS, TNFα, and nitric oxide (NO) (Izeboud et al., 1999; Sun et al., 2013), which further supports the interpretation of our results.

Mitochondrial dysfunction, as well as oxidative damage, are typical features observed in neurodegeneration including PD, which not only decline ATP production, but also increases ROS generation and induction of apoptosis. Mutations in genes that maintain mitochondrial quality control and function have been suggested as the main culprit that contributes toward familial PD (McLelland et al., 2014; Kazlauskaite and Muqit, 2015). Moreover, ROS production in neuronal cells (due to increase in ATP demand) is an important factor that leads to the demise of dopaminergic neurons. Mg^2+^ is also essential for ATP production and dysregulation of mitochondrial Mg^2+^ homeostasis has been shown to disrupt ATP production *via* the shift of mitochondrial energy metabolism and morphology (Romani, 2011; Zhang et al., 2012). Our results further provide evidence and we show for the first time that β-AR agonists activate TRPM7, which modulate Mg^2+^ homeostasis that prevents neurotoxin-induced loss of neuroblastoma cells. However, as these studies are mainly performed in isolated cells, they need to be replicated using dopaminergic cells and tissues. Nevertheless, these results identify the mechanisms involved in β-AR agonist induced protection of dopaminergic neurons by modulating TRPM7 expression thereby contributing to the survival of DA neurons.

## Data Availability Statement

The raw data supporting the conclusions of this manuscript will be made available by the authors, without undue reservation, to any qualified researcher.

## Author Contributions

BS and AK designed the studies that were performed by YS. YS and BS wrote the manuscript. AK edited the manuscript. All authors reviewed the results and approved the final manuscript.

## Conflict of Interest

The authors declare that the research was conducted in the absence of any commercial or financial relationships that could be construed as a potential conflict of interest.
